# Bacterial Community Dynamics and Ecological Characteristics in Temporary Holding Water of Live Seafood Tanks

**DOI:** 10.3390/biology15161355

**Published:** 2026-08-10

**Authors:** Ding Li, Yulin Lu, Jun Yuan, Xiaoping Wu, Weiqing Huang

**Affiliations:** 1 College of Marine Sciences, Ningde Normal University, Ningde 352100, Chinaluyulin0925@163.com (Y.L.); 2 Fujian Provincial University Engineering Research Center for Deep Processing of Mindong Aquatic Products, Ningde 352100, China; 3 School of Life Sciences, Fujian Agriculture and Forestry University, Fuzhou 350002, China; yjmail2008@fafu.edu.cn

**Keywords:** aquatic microenvironment, 16S rRNA gene sequencing, temporary holding water, bacterial community

## Abstract

Temporary holding water in live aquatic-product markets represents a short-term, animal-associated aquatic microenvironment, but its bacterial community dynamics remain poorly understood. In this study, 16S rRNA gene amplicon sequencing was used to characterize bacterial communities in holding water of *Anguilla japonica*, *Sinonovacula constricta*, and *Ruditapes philippinarum*. Samples were collected in the morning and evening to evaluate taxon-associated differences and short-term temporal shifts. The bacterial communities showed clear differentiation among the six animal–time groups. *Anguilla japonica* holding water showed distinct bacterial assemblages from the two bivalve-associated systems, whereas *S. constricta* and *R. philippinarum* holding water exhibited more pronounced morning-to-evening changes. In particular, *R. philippinarum* holding water had higher bacterial richness and a larger proportion of group-specific taxa in the morning, suggesting greater initial community heterogeneity. In addition, the occurrence–abundance analysis showed that taxa with broader distributions tended to have higher mean relative abundances, whereas Rud_8 contained a comparatively large proportion of low-occurrence taxa. Overall, this study demonstrates that live aquatic-product holding water harbors taxon-specific and time-responsive bacterial communities. These findings provide preliminary ecological insight into microbial dynamics in market-associated artificial aquatic microenvironments and highlight the need to consider aquatic animal type and sampling time in future water-quality monitoring.

## Simple Summary

Live seafood is commonly maintained in temporary holding tanks before sale, but the bacterial communities in the holding water may vary according to the aquatic animal being held and the sampling time. In this study, we examined bacterial communities in holding water from tanks containing *Anguilla japonica*, *Sinonovacula constricta*, and *Ruditapes philippinarum* in a retail market. Water samples were collected in the morning and evening from three independent tanks for each species, resulting in 18 samples. DNA-based bacterial community analysis showed clear differences among the six animal–time groups. *Anguilla japonica* holding water contained relatively high abundances of *Chryseobacterium* and *Flavobacterium*, whereas the bivalve-associated tanks showed marked morning-to-evening changes. Morning *R. philippinarum* holding water had the highest bacterial richness and the largest number of group-specific taxa. Because this study was based on 16S rRNA gene relative-abundance data and did not include water physicochemical measurements or species-level pathogen identification, the observed patterns should be interpreted as taxonomic associations rather than confirmed ecological functions or safety risks. These findings provide baseline information for future studies of microbial changes in live-seafood holding systems.

## 1. Introduction

Microbial communities are fundamental components of aquatic environments and play important roles in organic matter transformation, nutrient cycling, water-quality regulation, and ecosystem functioning [1,2,3]. In aquatic ecosystems, microorganisms are not only passive inhabitants of the water column, but also key biological drivers of carbon, nitrogen, and sulfur cycling [2,3]. Because bacterial assemblages can respond rapidly to environmental variation, substrate availability, animal-derived inputs, and human disturbance, microbial community structure is widely used to understand ecological processes and environmental changes in aquatic systems [3,4].

Temporary holding tanks in live aquatic-product markets represent a distinct but relatively understudied artificial aquatic microenvironment. Unlike natural rivers, lakes, estuaries, or aquaculture ponds, these systems are usually characterized by short-term water retention, high animal density, frequent human disturbance, and continuous biological inputs from live aquatic animals. Similar artificial aquatic systems, such as fish-holding tanks and aquaculture housing systems, have been shown to harbor distinct water microbiomes that can vary among tanks and change over time after animal introduction [5,6]. During temporary holding, microorganisms may be introduced into the surrounding water through animal mucus, feces, excretory products, body surfaces, and residual transport water. Fish mucus-associated microbiota form an important interface between the host and the surrounding water environment, and microbial communities associated with aquatic animals can differ substantially among body sites and host conditions [7,8]. At the same time, holding-water microbiota may be affected by species-specific physiological traits, feeding behavior, body surface characteristics, and short-term changes in the tank environment. This is particularly relevant for bivalves, which continuously filter suspended particles and microorganisms from surrounding water and can modify microbial and particulate dynamics through filtration, biodeposition, and excretion [9,10,11]. These features make market holding tanks useful small-scale systems for examining microbial community dynamics in artificial aquatic environments.

Previous studies have shown that aquatic animal-associated microbiota and surrounding water microbiota are closely linked, and that environmental factors can influence both host-associated and water microbial communities in aquaculture and shellfish systems [8,12]. In aquatic-product-related research, microbial studies have mainly focused on aquaculture systems, aquatic products, processing environments, spoilage organisms, or food safety risks [13,14]. By comparison, less attention has been paid to bacterial communities inhabiting temporary holding water in retail markets, despite the fact that this water directly contacts live aquatic products before purchase and may act as an interface for microbial exchange among animals, water, and market-associated disturbances. In addition, different aquatic animal taxa may influence holding-water microbiota in different ways. For example, fish-like and bivalve-associated systems differ in body structure, excretion pattern, habitat origin, and feeding behavior, while bivalves continuously interact with surrounding water through filter-feeding activity [9].

In this study, we did not aim merely to determine whether bacteria were present in live aquatic-product holding water. Instead, we focused on whether different aquatic animal taxa and short-term holding from morning to evening were associated with distinct bacterial community patterns. Three common market aquatic animals, including *Anguilla japonica* (Ang), *Sinonovacula constricta* (Sin), and *Ruditapes philippinarum* (Rud), were selected from temporary holding tanks. Water samples were collected at 08:00 and 18:00, with three biological replicates for each group. Using 16S rRNA gene amplicon sequencing, this study aimed to (i) characterize bacterial diversity and taxonomic composition in different holding-water systems; (ii) evaluate whether aquatic animal taxon and sampling time were associated with differences in bacterial community structure; (iii) identify dominant and differentially distributed bacterial genera among groups; and (iv) assess the prevalence–abundance relationship and persistence patterns. This study provides preliminary ecological information on microbial community dynamics in live aquatic-product holding water and may support future water-quality monitoring in retail market environments.

## 2. Materials and Methods

### 2.1. Sample Collection

Water samples were collected from temporary live aquatic-product holding tanks in a retail market located in Ningde, Fujian, China. Three aquatic animal taxa were selected, including *Anguilla japonica* (Ang), *Sinonovacula constricta* (Sin), and *Ruditapes philippinarum* (Rud). For each aquatic animal taxon, three physically independent holding tanks were selected, and each tank was treated as one biological replicate. Each tank contained approximately 7–10 individuals of the corresponding aquatic animal.

The same three independent tanks were sampled at two time points on the same day, at 08:00 and 18:00. Therefore, the morning and evening samples represented paired observations from the same holding tanks rather than independent tanks at each sampling time. The tanks were equipped with aeration devices but had no filtration systems. No water exchange or feeding was conducted between the two sampling times. The exact working water volume of each tank was not recorded. *A. japonica* was maintained in freshwater, whereas *S. constricta* and *R. philippinarum* were maintained in seawater supplied by the retailer.

The sample groups were designated as Ang_8, Ang_18, Sin_8, Sin_18, Rud_8, and Rud_18. Samples Ang1–Ang3, Sin1–Sin3, and Rud1–Rud3 represented the samples collected at 08:00, whereas Ang4–Ang6, Sin4–Sin6, and Rud4–Rud6 represented the corresponding samples collected at 18:00. This sampling design resulted in a total of 18 water samples, comprising three aquatic animal taxa × three independent holding tanks × two sampling times. For each sample, 1 L of holding water was collected using a sterile container and immediately transported to the laboratory on ice. Microbial biomass was collected by filtration through a sterile 0.22 μm membrane filter (Jinteng, Fuzhou, China), and the filters were stored at −80 °C until DNA extraction.

### 2.2. DNA Extraction, PCR Amplification, and Sequencing

Microbial genomic DNA was extracted directly from the 0.22 μm membrane filters obtained from each 1 L holding-water sample using the DNeasy PowerSoil Pro Kit, following the manufacturer’s instructions (QIAGEN, GmbH, Hilden, Germany). The concentration of the extracted DNA was measured using a Qubit 3.0 Fluorometer (Life Technologies, Thermo Fisher Scientific, Waltham, MA, USA) and the Equalbit 1× dsDNA HS Assay Kit (Vazyme, Nanjing, China). DNA quality and concentration were further evaluated using 1.0% agarose gel electrophoresis and a NanoDrop ND-2000 spectrophotometer (Thermo Scientific, Waltham, MA, USA).

The primer set 515FmodF (5′-GTGYCAGCMGCCGCGGTAA-3′) and 806R (5′-GGACTACHVGGGTWTCTAAT-3′) was used to amplify the V4 region of prokaryotic 16S rRNA genes, covering bacterial and archaeal lineages where amplified. PCR was conducted in a 20 μL reaction volume, including 10 ng of DNA, 0.8 μL of each primer (10 μM), 4 μL 5× FastPfu buffer (Vazyme, Nanjing, China), 0.4 μL FastPfu polymerase (Vazyme, Nanjing, China), 0.2 μL BSA (Vazyme, Nanjing, China), and 250 μM dNTPs (Vazyme, Nanjing, China). PCR cycling conditions were as follows: initial denaturation at 95 °C for 3 min, followed by 29 cycles of denaturation at 95 °C for 30 s, annealing at 55 °C for 30 s, extension at 72 °C for 45 s, and a final extension at 72 °C for 10 min.

Purified amplicons were combined in equimolar proportions and sequenced using an Illumina MiSeq PE300 platform, following the standard protocols provided by Majorbio Bio-Pharm Technology Co., Ltd. (Shanghai, China).

### 2.3. Sequence Processing and Taxonomic Annotation

Raw sequencing reads were demultiplexed according to sample-specific barcodes and processed using QIIME 2 [15]. Raw paired-end sequences were processed using the DADA2 plugin in QIIME 2 with the default parameters. No bases were trimmed from the 5′ ends of the forward or reverse reads (trim − left − f = 0 and trim − left − r = 0), and no fixed-length truncation was applied (trunc − len − f = 0 and trunc − len − r = 0). Reads with more than two expected errors in either the forward or reverse direction were discarded (max − ee − f = 2.0 and max − ee − r = 2.0). Reads were truncated at the first position with a quality score ≤ 2 (trunc − q = 2). Paired-end reads were merged using a minimum overlap of 12 bp, with no mismatches allowed in the overlapping region. Samples were denoised independently, and chimeric sequences were identified and removed using the consensus method. The resulting amplicon sequence variants were used for downstream analyses [16]. Representative ASV sequences were taxonomically classified against the SILVA database [17]. ASVs assigned to chloroplasts, mitochondria, eukaryotes, or unclassified non-target sequences were removed before downstream analyses. Community ecological analyses, including diversity analysis, ordination analysis, PERMANOVA, and multivariate-dispersion analysis, were conducted using the vegan package (version 2.6-8).

### 2.4. Alpha and Beta Diversity Analyses

To minimize the influence of unequal sequencing depth among samples, the denoised ASV table was rarefied to 36,968 sequences per sample, corresponding to the minimum number of denoised sequences among the 18 samples. All samples were retained after rarefaction. The rarefied ASV table was used to calculate alpha-diversity indices and generate rarefaction curves. Beta diversity was evaluated using Bray–Curtis dissimilarities calculated from the ASV-level relative-abundance table.

Alpha diversity was evaluated using Chao, Shannon, and Coverage indices. Rarefaction curves were generated to assess whether sequencing depth was sufficient to capture bacterial diversity across samples. Differences in bacterial richness and diversity among groups were visualized based on the six sample groups: Ang_8, Ang_18, Sin_8, Sin_18, Rud_8, and Rud_18.

Beta diversity was evaluated using Bray–Curtis dissimilarities calculated from the ASV-level relative-abundance table. Principal coordinate analysis (PCoA) was performed to visualize variation in bacterial community structure among samples. Overall differences among the six taxon–time groups—Ang_8, Ang_18, Sin_8, Sin_18, Rud_8, and Rud_18—were evaluated using one-factor permutational multivariate analysis of variance (PERMANOVA) implemented with the adonis2 function in the vegan package, with 999 permutations. Homogeneity of multivariate dispersion among the six groups was assessed using the betadisper function based on the same Bray–Curtis dissimilarity matrix, followed by a permutation test with 999 permutations. The pseudo-F statistic, R^2^ effect size, and permutation-based *p*-value were reported.

### 2.5. Taxonomic Composition and Differential Taxa Analysis

Relative abundances of bacterial taxa were summarized at the phylum, family, and genus levels. Stacked bar plots were used to show the dominant bacterial taxa in each sample. Differences in genus-level relative abundances among the six holding-water groups were evaluated using the Kruskal–Wallis rank-sum test. All genus-level taxonomic features, including unclassified taxa, were retained in the analysis. *p*-values were adjusted for multiple comparisons using the false-discovery-rate procedure, and an FDR-adjusted *p*-value < 0.05 was considered statistically significant. Pairwise post hoc comparisons were performed using the Tukey–Kramer test at a 95% confidence level.

### 2.6. Shared and Unique Taxa Analysis

Venn analysis was performed to compare shared and unique bacterial taxa among the six groups at both the ASV and genus levels. The number and proportion of unique taxa in each group, as well as shared taxa among all groups, were calculated to evaluate the common and group-specific bacterial components in different holding-water systems.

### 2.7. Occurrence–Abundance Relationship and Occurrence-Frequency Classification

The occurrence frequency and mean relative abundance of bacterial taxa were calculated across all 18 samples. Linear regression analysis was used to evaluate the relationship between taxon occurrence frequency and mean relative abundance. Taxa were operationally classified as low-occurrence taxa when detected in less than 20% of samples, intermediate-occurrence taxa when detected in 20–80% of samples, and high-occurrence taxa when detected in more than 80% of samples. These categories describe prevalence across the sampled holding-water systems and do not necessarily indicate temporal persistence, physiological resistance, active growth, or continuous input from aquatic animals.

### 2.8. Statistical Analysis

All statistical analyses were performed in R version 4.3.2. Community ecological analyses, including diversity analysis, ordination analysis, PERMANOVA, and multivariate-dispersion analysis, were conducted using the vegan package [18]. Data visualization was performed using ggplot2 (version 4.0.3), pheatmap (version 1.0.13), and VennDiagram (version 1.8.2) [19]. Unless otherwise stated, *p* < 0.05 was considered statistically significant.

### 2.9. Nucleotide Accession Numbers

The raw sequence data are available in the Genome Sequence Archive (Genomics, Proteomics & Bioinformatics 2021) at the National Genomics Data Center (Nucleic Acids Res 2022), China National Center for Bioinformation/Beijing Institute of Genomics, Chinese Academy of Sciences (accession codes: CRA043346), and can be accessed at https://ngdc.cncb.ac.cn/gsa, accessed on 31 July 2026. Data availability for peer review: CRA043346 (https://ngdc.cncb.ac.cn/gsa/s/u3rVm47K, accessed on 31 July 2026), the data has been released.

## 3. Results

### 3.1. Sequencing Depth and Alpha-Diversity Patterns

Following DADA2 quality filtering, denoising, paired-end merging, and chimera removal, a total of 836,936 denoised sequences were retained across the 18 samples. The number of retained sequences ranged from 36,968 to 111,881 per sample, with a mean of 46,496 sequences per sample. The rarefaction curves gradually approached saturation, and the coverage values approached 1.0, indicating that the sequencing depth was adequate for the subsequent bacterial community analyses (Supplementary Figure S1).

### 3.2. Bacterial Community Structure Based on PCoA

**Figure 1:**
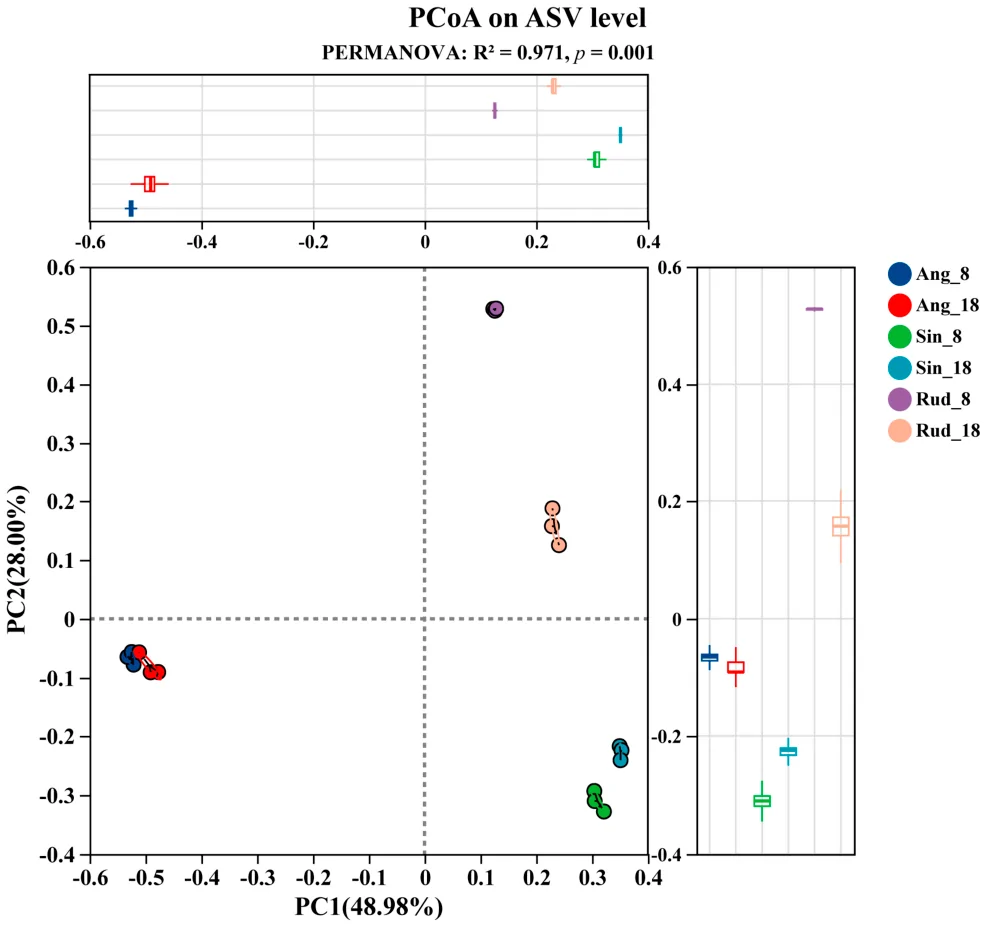
Principal coordinate analysis of bacterial community structure in temporary holding water based on Bray–Curtis dissimilarities. Different colors indicate the six holding-water groups: Ang_8, Ang_18, Sin_8, Sin_18, Rud_8, and Rud_18. PERMANOVA indicated significant differences among the six groups (pseudo-F = 80.381, R^2^ = 0.971, *p* = 0.001), whereas multivariate dispersion did not differ significantly among groups (F = 1.255, *p* = 0.359).

Principal coordinate analysis based on Bray–Curtis dissimilarities showed clear differentiation among the six holding-water groups (Figure 1). The first two principal coordinate axes explained 48.98% and 28.00% of the total variation, respectively, together accounting for 76.98% of the variation in bacterial community structure. PERMANOVA indicated significant differences in bacterial community composition among the six taxon–time groups (pseudo-F = 80.381, R^2^ = 0.971, *p* = 0.001), with group identity explaining 97.10% of the total community variation. The test for homogeneity of multivariate dispersion was not significant (F = 1.255, *p* = 0.359), indicating that the PERMANOVA result was unlikely to be primarily driven by unequal within-group dispersion. Ang_8 and Ang_18 were positioned relatively close to each other, whereas the morning and evening samples of Sin and Rud occupied different regions of the ordination space. Because the six taxon–time combinations were analyzed as a single grouping factor, the independent contributions of aquatic animal taxon and sampling time were not quantified separately.

### 3.3. Taxonomic Composition of Bacterial Communities

**Figure 2:**
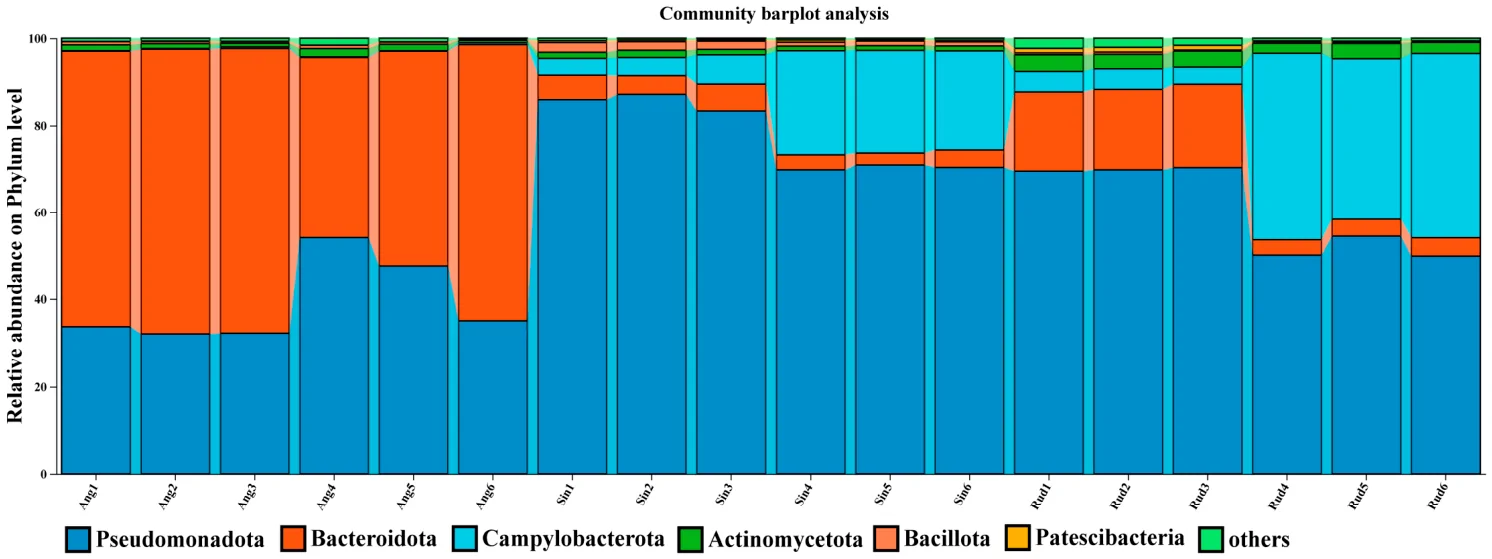
Taxonomic composition at phylum levels. Samples Ang1–Ang3, Sin1–Sin3, and Rud1–Rud3 represent morning samples collected at 08:00, whereas Ang4–Ang6, Sin4–Sin6, and Rud4–Rud6 represent evening samples collected at 18:00. Taxa with low relative abundance were grouped as “others”.

At the phylum level, bacterial communities were mainly composed of *Pseudomonadota*, *Bacteroidota*, *Campylobacterota*, *Actinomycetota*, *Bacillota*, and *Patescibacteria*, but their relative abundances differed markedly among aquatic animal taxa and sampling times (Figure 2). Ang samples contained a high proportion of *Bacteroidota*, especially in Ang_8 samples, whereas Sin and Rud samples were generally dominated by *Pseudomonadota*. *Campylobacterota* showed a clear increase in several evening bivalve-associated holding-water samples, particularly in Sin_18 and Rud_18, suggesting a time-associated shift in bacterial composition in bivalve-associated holding water.

At the family level, the dominant bacterial families differed among the three groups (Supplementary Figure S2). Ang samples were characterized by relatively high proportions of *Weeksellaceae*, *Flavobacteriaceae*, and *Comamonadaceae*. Sin samples were mainly dominated by *Moraxellaceae* in the morning, while *Arcobacteraceae* increased in the evening. Rud samples showed a more complex family-level composition in the morning, with contributions from *Pseudoalteromonadaceae*, *Vibrionaceae*, Clade_I, *Colwelliaceae*, and other families. In the evening, Rud samples showed increased proportions of *Moraxellaceae* and *Arcobacteraceae*.

**Figure 3:**
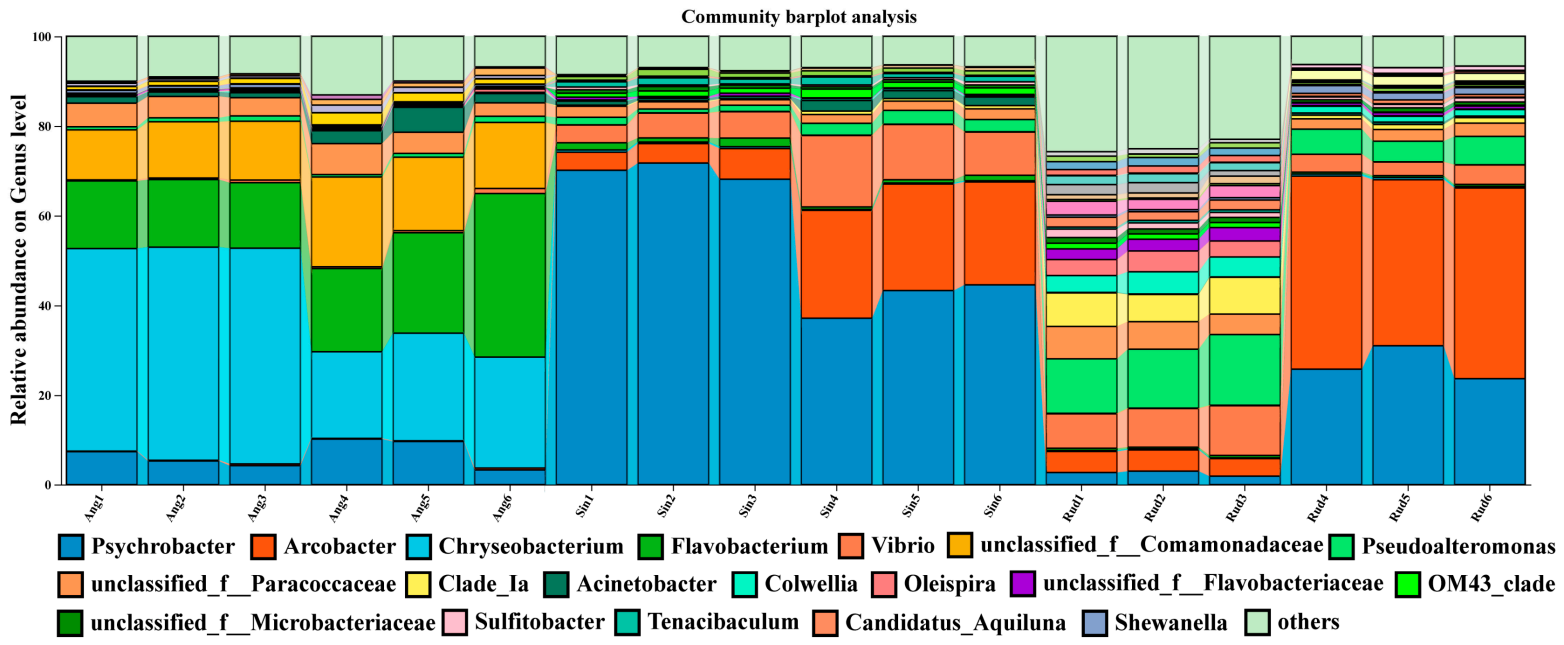
Taxonomic composition at genus levels. Samples Ang1–Ang3, Sin1–Sin3, and Rud1–Rud3 represent morning samples collected at 08:00, whereas Ang4–Ang6, Sin4–Sin6, and Rud4–Rud6 represent evening samples collected at 18:00. Taxa with low relative abundance were grouped as “others”.

At the genus level, the bacterial community profiles showed stronger taxon-specific and time-associated patterns (Figure 3). Ang samples were characterized by high relative abundances of *Chryseobacterium* and *Flavobacterium*, with *Chryseobacterium* being particularly abundant in Ang_8 samples. Sin_8 samples were strongly dominated by *Psychrobacter*, whereas Sin_18 samples showed an increased contribution of *Arcobacter* and *Vibrio*. Rud_8 samples displayed a more diverse genus-level structure, with relatively high proportions of *Pseudoalteromonas*, *Vibrio*, Clade_Ia, *Colwellia*, and *Oleispira*. By contrast, Rud_18 samples were dominated mainly by *Arcobacter* and *Psychrobacter*. These results indicate that *Anguilla japonica*, *S. constricta*, and *R. philippinarum* holding waters maintained distinct bacterial signatures, and that the bivalve-associated holding-water communities underwent clear morning-to-evening compositional shifts.

### 3.4. Differentially Distributed Bacterial Genera Among Holding-Water Groups

**Figure 4:**
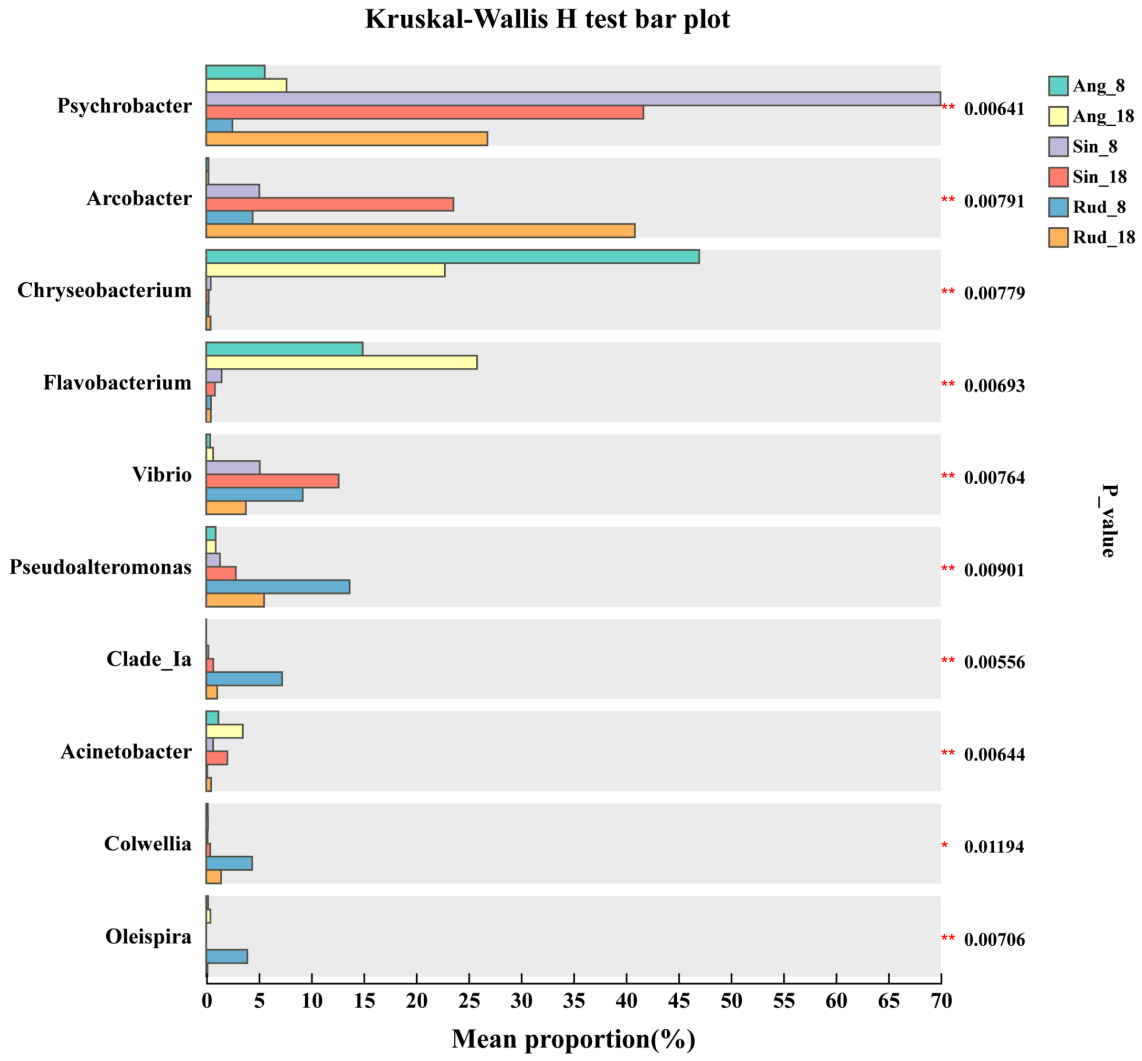
Differentially distributed bacterial genera among the six holding-water groups. Bars represent the mean relative abundance of each genus in each group. Asterisks indicate significant differences among groups.

The Kruskal–Wallis H test identified several bacterial genera that differed significantly among the six holding-water groups (Figure 4). The significantly different genera included *Psychrobacter*, *Arcobacter*, *Chryseobacterium*, *Flavobacterium*, *Vibrio*, *Pseudoalteromonas*, Clade_Ia, *Acinetobacter*, *Colwellia*, and *Oleispira*. Among these, *Psychrobacter* showed the highest mean relative abundance in Sin_8 and remained abundant in some evening bivalve-associated samples. *Arcobacter* was mainly enriched in Sin_18 and Rud_18, indicating a stronger association with evening bivalve-associated holding water. *Chryseobacterium* and *Flavobacterium* were mainly enriched in Ang samples, especially in Ang_8 and Ang_18. *Pseudoalteromonas*, *Colwellia*, and *Oleispira* were more characteristic of Rud_8 samples, consistent with the more diverse community structure observed in *R. philippinarum* morning holding water. These differentially distributed genera further support the presence of taxon-specific and time-associated microbial patterns in temporary holding tanks.

### 3.5. Shared and Unique Bacterial Taxa Among Groups

Venn analysis was performed to compare shared and unique bacterial components among the six groups at the genus and ASV levels (Supplementary Figure S2). At the ASV level, only 42 ASVs were shared by all groups, accounting for 2.00% of the total ASVs. Rud_8 contained the highest number of unique ASVs, with 545 unique ASVs accounting for 25.89% of the total ASVs. At the genus level, 54 genera were shared by all groups, accounting for 8.91% of the total detected genera. Rud_8 also contained the largest number of unique genera, with 84 unique genera accounting for 13.86% of the total genus-level taxa. These results indicate that although a small shared bacterial pool was present across all holding-water systems, each group also contained distinct bacterial components, with Rud_8 showing the strongest uniqueness and highest taxonomic richness (Supplementary Figure S3).

### 3.6. Occurrence–Abundance Relationship and Occurrence-Frequency Patterns

**Figure 5:**
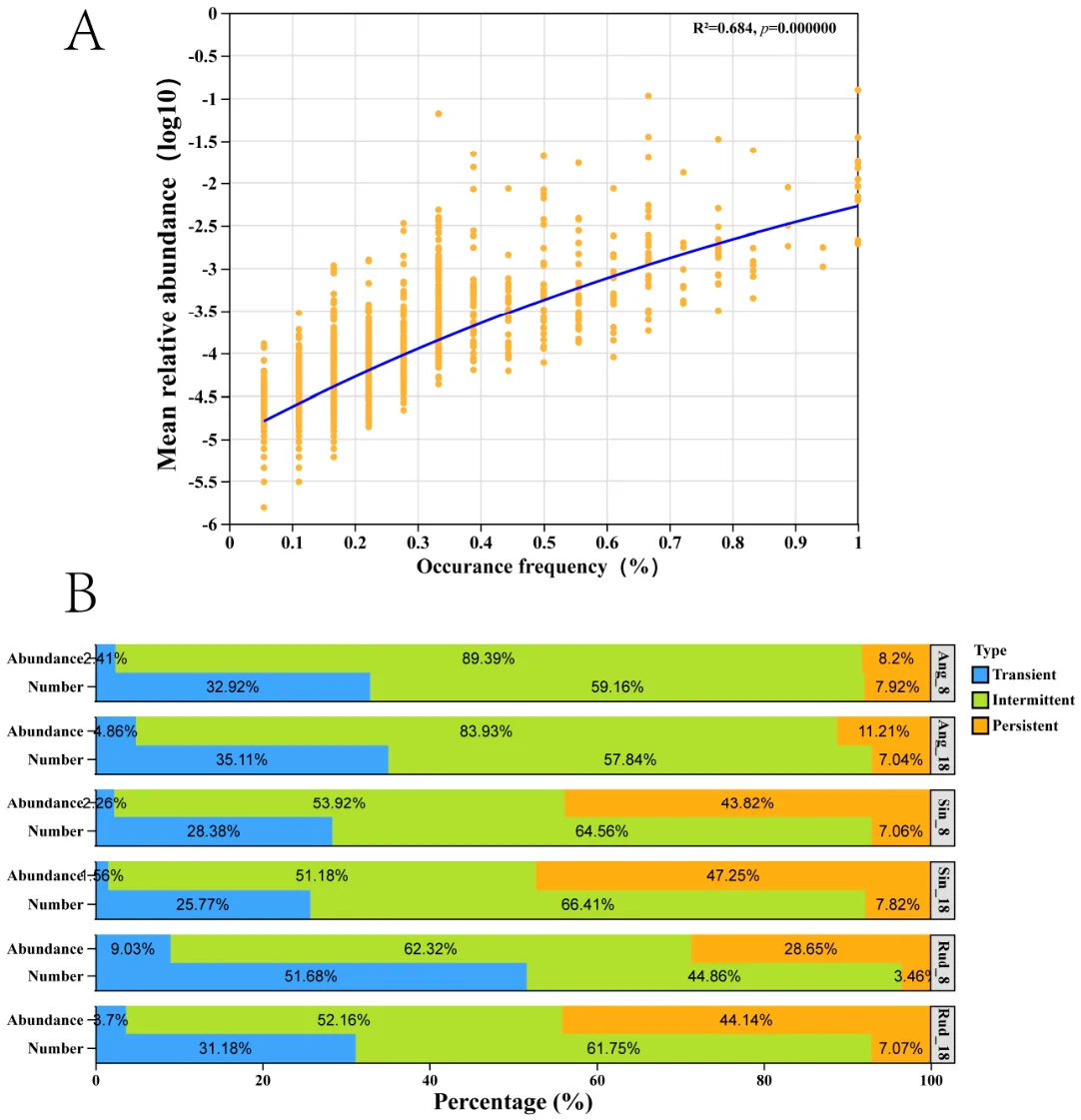
Occurrence–abundance relationship and occurrence-frequency categories of bacterial taxa in temporary holding water. (**A**) Relationship between occurrence frequency and mean relative abundance across all samples. (**B**) Relative contributions of low-occurrence, intermediate-occurrence, and high-occurrence taxa in each holding-water group based on taxon number and relative abundance.

The occurrence frequency of bacterial taxa was positively associated with their mean relative abundance (R^2^ = 0.684, *p* < 0.001; Figure 5A). Taxa with higher occurrence frequency tended to have higher mean relative abundance, suggesting that widely distributed taxa also contributed more strongly to the total bacterial community. This pattern indicates that the holding-water microbiota was structured not only by rare and low-occurrence taxa but also by a subset of prevalent and relatively abundant bacterial members.

To further characterize ecological distribution patterns, bacterial taxa were classified into transient, intermittent, and persistent categories based on their occurrence patterns (Figure 5B). In terms of taxon number, intermediate-occurrence taxa accounted for the largest proportion in Ang_8, Ang_18, Sin_8, Sin_18, and Rud_18, while low-occurrence taxa were particularly abundant in Rud_8. In terms of relative abundance, Ang_8 and Ang_18 were strongly dominated by intermediate-occurrence taxa, which accounted for 89.39% and 83.93% of total relative abundance, respectively. In contrast, high-occurrence taxa contributed substantially to the relative abundance of bivalve-associated holding-water communities, especially in Sin_8, Sin_18, and Rud_18, where they accounted for 43.82%, 47.25%, and 44.14% of total relative abundance, respectively. These results suggest that Anguilla japonica holding water was mainly dominated by intermittent bacterial members, whereas bivalve-associated holding water contained a larger contribution from persistent and highly abundant taxa. These categories describe differences in prevalence across the 18 samples rather than temporal persistence within individual tanks.

## 4. Discussion

### 4.1. Temporary Holding Water Represents a Distinct Short-Term Artificial Aquatic Microenvironment

Temporary holding tanks in live aquatic-product markets represent a distinctive artificial aquatic microenvironment characterized by short water-retention time, high animal density, continuous animal-derived inputs, and frequent disturbance. Unlike conventional aquaculture systems designed for long-term production, these tanks operate over a period of hours and may therefore capture early microbial restructuring at the water–animal interface. The taxon-associated and time-responsive patterns observed in this study indicate that even such transient systems can retain reproducible ecological organization rather than functioning as microbiologically homogeneous storage water. Interactions among water, biofilm, feed, and animal-associated microbiota have been documented in engineered aquaculture systems, but temporary retail holding water has received much less attention [6,7,20]. We therefore propose that retail holding tanks can serve as small-scale systems for examining how microbial source inputs and short-term environmental filtering jointly shape aquatic bacterial communities [21]. This perspective extends microbial research from long-term aquaculture environments to short-duration market-associated systems and provides the ecological context for the taxonomic and temporal patterns discussed below.

### 4.2. Distinct Bacterial Assemblages Among the Three Holding-Water Systems

Because the present study was based on genus-level relative-abundance patterns obtained through 16S rRNA gene amplicon sequencing, the ecological interpretations below should be regarded as hypotheses informed by previously reported habitat associations. These patterns do not demonstrate metabolic activity, substrate utilization, competitive ability, environmental adaptation, pathogenicity, or direct ecological function. A major finding of this study was that the three holding-water systems harbored distinct bacterial assemblages. However, an important confounding factor should be considered: *A. japonica* was maintained in freshwater, whereas *S. constricta* and *R. philippinarum* were maintained in seawater. Therefore, the pronounced differentiation between the Ang groups and the two bivalve-associated groups may partly, or potentially largely, reflect differences in water source, salinity, and associated physicochemical conditions rather than aquatic animal taxon alone. Because aquatic animal taxon and water type were not independently manipulated in the present design, their individual contributions cannot be disentangled. Accordingly, the observed differences should be interpreted as bacterial community patterns associated with distinct animal–holding-water systems rather than as effects attributable solely to aquatic animal identity. Within this constraint, animal-associated microbial inputs and animal-specific interactions with the surrounding water may also have contributed to the observed community patterns.

The *A. japonica* holding water contained higher relative abundances of *Bacteroidota*, *Weeksellaceae*, *Flavobacteriaceae*, *Chryseobacterium*, and *Flavobacterium*. Members of *Flavobacteriaceae* are commonly reported from aquatic environments and have previously been associated with fish surfaces, organic particles, mucosal habitats, and polymer-rich substrates [22]. These reported habitat associations provide possible ecological context for the taxonomic pattern observed in the Ang samples. However, the present data do not demonstrate that *Chryseobacterium* or *Flavobacterium* utilized animal-derived polymers, mucus-associated substrates, or dissolved organic matter. These genera should therefore be interpreted as taxonomic features associated with the *A. japonica* holding-water environment rather than as confirmed functional or pathogenic agents.

The *S. constricta* holding water was strongly characterized by *Psychrobacter*, especially in Sin_8. *Psychrobacter* is frequently reported in marine, aquatic-product, and seafood-related environments and is often associated with cold-tolerant or nutrient-responsive bacterial assemblages [13]. The high relative abundance of *Psychrobacter* in Sin_8 represented a distinct taxonomic feature of the morning *S. constricta* holding-water community. Its lower relative abundance in the evening was accompanied by increased relative abundances of *Arcobacter* and other taxa, indicating short-term compositional restructuring. However, the present relative-abundance data cannot determine whether this pattern resulted from active bacterial growth, continuous animal-derived input, loss of other taxa, or unmeasured changes in the holding-water environment.

The *R. philippinarum* holding water was particularly notable because Rud_8 showed both the highest richness and the largest number of unique ASVs and genera. This suggests that the morning *R. philippinarum* holding water contained a more heterogeneous microbial pool than the other groups. The comparatively high relative abundances of *Pseudoalteromonas*, *Vibrio*, Clade_Ia, *Colwellia*, and *Oleispira* in Rud_8 were consistent with a taxonomically heterogeneous assemblage containing several marine-associated genera. For example, *Pseudoalteromonas* has frequently been reported from marine surfaces, particles, biofilms, and higher organisms [23,24]. These previously reported habitat associations provide possible context for its occurrence in Rud_8, but the source and ecological activity of these bacteria were not determined in the present study. The subsequent increase in the relative abundances of *Arcobacter* and *Psychrobacter* in Rud_18 represents a change in community composition but does not demonstrate that these taxa were more competitive or better adapted to the evening holding conditions.

### 4.3. Bivalve-Associated Holding Water Showed Stronger Short-Term Restructuring than Anguilla Japonica Holding Water

The morning-to-evening comparison revealed that the two bivalve-associated systems showed more pronounced temporal shifts than the *A. japonica*-associated system. At the phylum level, *Campylobacterota* increased in Sin_18 and Rud_18. At finer taxonomic resolution, this shift was mainly reflected by the enrichment of *Arcobacteraceae* and *Arcobacter*. This consistent increase in both bivalve-associated systems suggests that the evening bacterial community was not merely a random variant of the morning community but represented a directional change in community composition.

The stronger morning-to-evening variation observed in the two bivalve-associated systems may be considered in the context of bivalve filtration, particle selection, biodeposition, feces and pseudofeces production, and excretion [9]. These processes have the potential to alter suspended particles, organic-matter availability, and the exchange of microorganisms between animals and surrounding water. However, these processes were not measured directly in the present study and should therefore be regarded as possible ecological explanations rather than demonstrated mechanisms. Unmeasured changes in water physicochemical conditions and market-management practices may also have contributed to the observed temporal patterns.

The increase in Arcobacter in Sin_18 and Rud_18 deserves careful interpretation. *Arcobacter* and related *Arcobacteraceae* have been reported from aquatic environments, shellfish, seafood, wastewater-impacted water, and other water-associated matrices [25,26]. Several species within this group are considered emerging food- or water-associated bacteria of potential public health relevance [25]. However, the present study detected *Arcobacter* at the 16S genus level only. This means that we cannot determine whether the sequences belong to pathogenic species, non-pathogenic environmental lineages, viable cells, or relic DNA. Therefore, the observed enrichment should not be described as direct evidence of pathogen accumulation. A more appropriate interpretation is that evening bivalve-associated holding water showed an increased relative abundance of an *Arcobacter*-affiliated bacterial group. This pattern may be considered a candidate taxonomic signal for future targeted monitoring, pending species-level identification, viability assessment, and pathogenicity validation.

The appearance of *Vibrio* among significantly different genera should be interpreted cautiously. *Vibrio* species are natural inhabitants of marine and estuarine environments, but the genus also includes seafood-associated species of food-safety concern, such as *V. parahaemolyticus* and *V. vulnificus* [27,28,29,30]. Because 16S rRNA sequencing does not provide reliable species-level resolution for many closely related taxa and cannot confirm virulence potential, the present result should be discussed as a community-level signal rather than as a diagnostic result. Future studies should combine 16S surveys with culture-based isolation, quantitative PCR targeting specific species or virulence genes, viability assays, and metagenomic sequencing to evaluate whether the observed genus-level patterns have practical implications for water quality or aquatic-product safety. However, because physicochemical variables were not measured concurrently, the observed morning-to-evening differences cannot be attributed to specific changes in temperature, salinity, dissolved oxygen, pH, inorganic nutrients, or organic-matter availability. Sampling time should therefore be interpreted as an integrative temporal factor associated with multiple unmeasured changes in the holding environment rather than as an independently demonstrated ecological driver.

### 4.4. Morning R. philippinarum Holding Water Represents a Highly Heterogeneous Initial Community State

The distinct position of Rud_8 in alpha diversity, Venn analysis, and taxonomic composition suggests that the morning *R. philippinarum* holding water represented a highly heterogeneous initial community state. Rud_8 had the highest Chao and Shannon values, the largest number of unique ASVs and genera, and a community containing multiple relatively abundant genera rather than a single overwhelmingly dominant taxon. This pattern is ecologically important because it indicates that higher diversity was associated with greater group-specificity rather than with a larger shared core community, which is consistent with the view that core and accessory microbiota may represent different ecological components of microbial communities [31,32].

One possible interpretation is that *R. philippinarum* introduced a broad range of microbial members into the holding water through shell surfaces, residual sediment particles, biodeposits, or original source water. Previous studies have shown that bivalves can harbor tissue- and organ-associated microbial communities, and that these communities are influenced by environmental sources such as surrounding water and sediment [33]. Because bivalves interact intensively with suspended particles through filter-feeding activity, they may selectively remove, concentrate, transform, and release microorganisms and particle-associated bacteria through filtration, biodeposition, and excretion processes [9,10,34]. The presence of *Pseudoalteromonas*, *Colwellia*, and *Oleispira* in Rud_8 is consistent with a community containing diverse marine-associated members, among which *Pseudoalteromonas* is well known for its associations with marine particles, surfaces, biofilms, and higher organisms [23,24]. However, the evening Rud community became much more dominated by *Arcobacter* and *Psychrobacter*, indicating that the initially heterogeneous community did not remain stable during short-term holding.

This transition may reflect a shift from an input-dominated community state to a selection-dominated community state. In the morning, the bacterial assemblage may still retain signals from the original animal-associated, sediment-associated, and transport-associated microbial pools. By the evening, taxa better adapted to the tank conditions, available organic substrates, or bivalve-modified water environment may increase in relative abundance. This interpretation is consistent with microbial community assembly theory, in which dispersal, environmental selection, ecological drift, and diversification jointly shape community composition across spatial and temporal scales [21,35]. However, the present study did not directly quantify these sources or distinguish among dispersal, environmental selection, bacterial growth, mortality, and compositional replacement. Therefore, the taxa showing higher relative abundances in Rud_18 should not be interpreted as being demonstrably more competitive or better adapted to the holding-water conditions. Likewise, the lower occurrence of some taxa in the full dataset does not demonstrate their disappearance or inability to persist within individual tanks. Source tracking, absolute quantification, metagenomic or metatranscriptomic analyses, and cultivation-based validation would be required to test these ecological mechanisms.

It should be emphasized that the occurrence-frequency categories used in this study describe taxon prevalence across the 18 samples rather than temporal persistence within an individual holding tank. High-occurrence taxa, defined as taxa detected in more than 80% of the samples, represent broadly distributed members of the sampled holding-water communities. Their broad distribution may result from repeated animal-associated input, tolerance of environmental conditions shared among tanks, or growth within the water; however, the present study design cannot distinguish among these mechanisms. In contrast, low-occurrence taxa may represent restricted, episodic, or source-specific microbial inputs. The relatively high proportion of low-occurrence taxa in Rud_8 is consistent with its greater richness and number of group-specific ASVs, suggesting that the morning *R. philippinarum* holding water retained a heterogeneous microbial pool derived from animals, residual sediment, source water, or transport-associated inputs. Therefore, occurrence frequency should not be interpreted as direct evidence of environmental resistance, active reproduction, or long-term temporal persistence.

### 4.5. Implications for Water-Quality Monitoring and Holding-Tank Management

The taxonomic patterns observed in this study can be used to prioritize monitoring targets, although they do not yet provide validated water-quality indicators. In particular, the evening enrichment of *Arcobacter*-affiliated sequences in both bivalve-associated systems and the differential distribution of *Vibrio* identify these groups as candidate surveillance signals for subsequent targeted assessment. However, because the present data are based on genus-level relative abundances, these taxa cannot be directly interpreted as pathogenic organisms or converted into numerical safety thresholds. Therefore, no universal abundance threshold can be proposed from the current dataset. A practical monitoring framework should combine standardized morning and evening microbial sampling with measurements of temperature, salinity, dissolved oxygen, pH, ammonium, nitrite, turbidity, and organic matter. Increases in *Arcobacter*- or *Vibrio*-affiliated groups should be confirmed using species-specific qPCR, cultivation, viability assays, or virulence-gene detection. When such microbial changes occur together with deteriorating physicochemical conditions, management measures such as shortening water-renewal intervals, improving aeration or filtration, removing accumulated biodeposits, and maintaining stable temperature should be considered. Reliable alert thresholds will require longitudinal sampling across multiple markets, absolute bacterial quantification, and validation against water-quality deterioration and aquatic-animal health outcomes.

### 4.6. Limitations and Future Perspectives

Several limitations should be acknowledged. First, this study included 18 samples from three aquatic animal taxa and two sampling times. Although this design provides a clear comparison among taxa and between morning and evening samples, it remains limited in temporal and spatial scope. Additional markets, seasons, management conditions, and repeated sampling days are needed to determine whether the observed patterns are generalizable. Second, the physicochemical characteristics of the holding water were not measured concurrently. Variables such as temperature, salinity, dissolved oxygen, pH, ammonium, nitrite, nitrate, turbidity, and dissolved organic carbon may change during short-term holding and can substantially influence bacterial community composition. Consequently, the observed morning-to-evening differences cannot be attributed to any specific environmental variable or to holding duration alone. In the present study, sampling time represents an integrative temporal factor that may encompass changes in animal activity, animal-derived inputs, microbial growth, water chemistry, and routine tank-management processes. Future studies should combine repeated microbial sampling with concurrent physicochemical measurements to identify the environmental factors associated with short-term bacterial community restructuring. Third, this study relied on relative-abundance 16S rRNA gene amplicon data. Such data are powerful for community profiling, but they are compositional and do not provide absolute bacterial loads [36]. An increase in the relative abundance of one genus may reflect true growth, decline in other taxa, or both. Therefore, future work should incorporate absolute quantification, such as flow cytometry, qPCR, or spike-in standards, to distinguish relative compositional shifts from changes in bacterial biomass. Fourth, although three physically independent holding tanks were included for each aquatic animal taxon, the exact working water volume and concurrent physicochemical characteristics of the tanks were not recorded. Therefore, potential variation in water source, salinity, temperature, dissolved oxygen, and other tank conditions may also have contributed to the observed bacterial differences. Fifth, in addition, aquatic animal taxon was confounded with water type because *A. japonica* was maintained in freshwater, whereas the two bivalve species were maintained in seawater. Consequently, the independent effects of animal identity, water source, and salinity could not be resolved in the present study. Finally, functional interpretation remains limited. Although the present study characterized taxonomic composition and occurrence patterns, it did not directly measure functional genes, metabolic activity, absolute bacterial abundance, or virulence potential. Future studies should integrate PICRUSt2 or FAPROTAX for preliminary functional inference, and preferably use metagenomics, metatranscriptomics, targeted functional gene qPCR, or cultivation to validate the ecological and practical significance of key bacterial taxa.

## 5. Conclusions

This study demonstrates that temporary holding water in live-seafood retail tanks is a structured, taxon-associated, and rapidly changing artificial aquatic microenvironment. Across the three aquatic animal taxa examined, animal-associated microbial inputs and short-term tank conditions jointly contributed to bacterial community differentiation, while the two bivalve-associated systems showed more pronounced morning-to-evening restructuring than the *Anguilla japonica* system. These findings suggest that microbial monitoring in retail holding systems should use standardized sampling times and should be combined with physicochemical measurements and targeted surveillance of groups such as *Arcobacter*- and *Vibrio*-affiliated bacteria, particularly in bivalve-associated tanks. However, numerical alert thresholds cannot be established from the present relative-abundance dataset and will require repeated multi-market sampling, absolute quantification, and species- or virulence-specific validation. Overall, these findings indicate that live aquatic-product holding water is a dynamic artificial aquatic microenvironment exhibiting taxon-associated and sampling-time-associated bacterial community patterns. Because physicochemical variables were not measured concurrently, the environmental mechanisms underlying these patterns could not be resolved. Future studies integrating water-quality measurements, absolute quantification, culture-based detection, qPCR, and metagenomic approaches are needed to identify the environmental drivers of community variation and validate potential risk-related implications.

## Data Availability

The original contributions presented in the study are included in the article/Supplementary Material. Further inquiries can be directed to the corresponding author.

## Supplementary Materials

The following supporting information can be downloaded at: https://www.mdpi.com/article/10.3390/biology15161355/s1, Figure S1: Rarefaction curves of bacterial communities in temporary holding water; Figure S2: Family-level composition of bacterial communities in temporary holding water; Figure S3: Shared and unique bacterial taxa among the six holding-water groups.
