# Supplementary material for: Bacterial Community Dynamics and Ecological Characteristics in Temporary Holding Water of Live Seafood Tanks
Source: Biology (Basel). 2026 Aug 10;15(16):1355. doi: 10.3390/biology15161355 (PMC13509187; doi:10.3390/biology15161355)
Supplement: Supplementary file 1 [file biology-15-01355-s001.zip › biology-4453999-supplementary.pdf]

biology

MDPI

Supplementary Material

Supplementary Figures

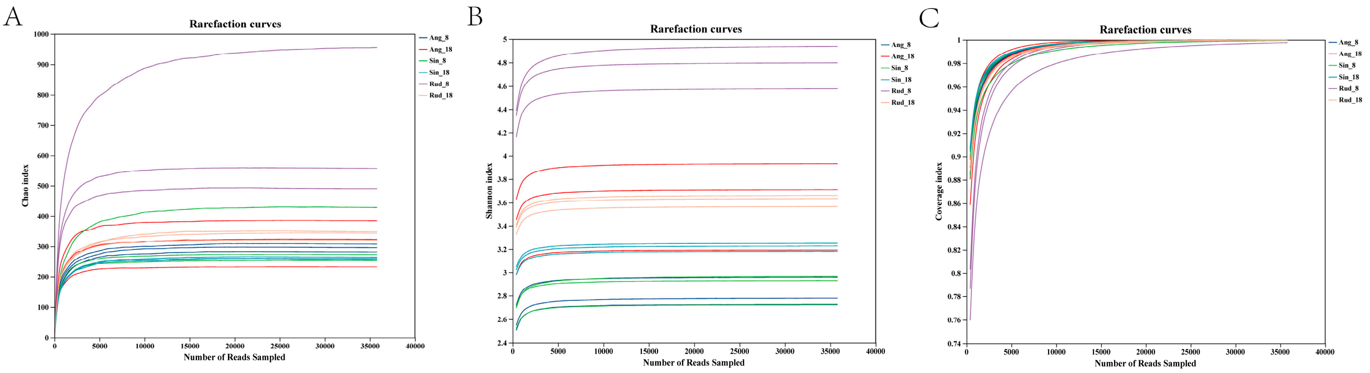

FIGURE S1 Rarefaction curves of bacterial communities in temporary holding water. Rarefaction curves based on the Chao index (A), Shannon index (B), and Coverage index (C) were used to evaluate sequencing depth and bacterial diversity across the six holding-water groups. Ang_8, Sin_8, and Rud_8 represent morning samples collected at 08:00 from *Anguilla japonica*, *Sinonovacula constricta*, and *Ruditapes philippinarum* holding water, respectively; Ang_18, Sin_18, and Rud_18 represent evening samples collected at 18:00.

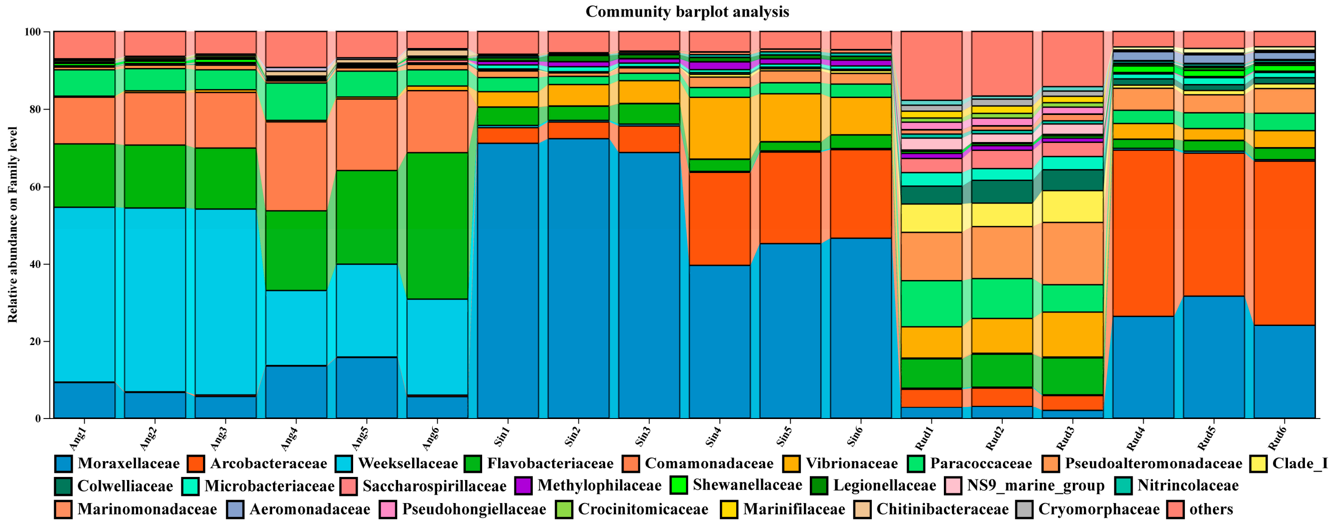

FIGURE S2 Family-level composition of bacterial communities in temporary holding water.

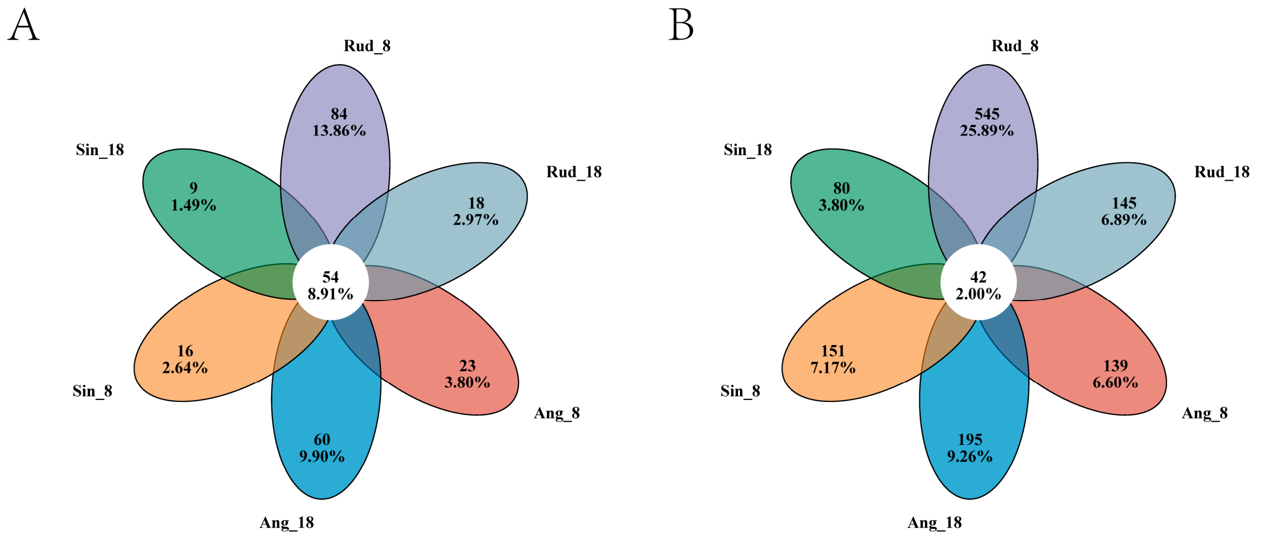

FIGURE S3 Shared and unique bacterial taxa among the six holding-water groups.
